# Factors influencing insulin initiation in primary care facilities in Cape Town, South Africa

**DOI:** 10.4102/safp.v65i1.5656

**Published:** 2023-02-28

**Authors:** Tasunungurwa T. Mathose, Robert Mash

**Affiliations:** 1 Division of Family Medicine and Primary Care, Faculty of Medicine and Health Sciences, Stellenbosch University, Cape Town, South Africa

**Keywords:** type 2 diabetes, insulin, primary healthcare, primary care, initiation, patient education and counselling

## Abstract

**Background:**

Type 2 diabetes (T2DM) is a leading cause of mortality in South Africa and resistance to the use of insulin is common. This study aimed to explore factors that influence the initiation of insulin in patients with T2DM in primary care facilities in Cape Town, South Africa.

**Methods:**

An exploratory descriptive qualitative study was conducted. Seventeen semi-structured interviews were held with patients eligible for insulin, on insulin and primary care providers. Participants were selected by maximum variation purposive sampling. Data were analysed using the framework method in Atlas-ti.

**Results:**

Factors related to the health system, service delivery, clinical care and patients. Systemic issues related to the required inputs of workforce, educational materials, and supplies. Service delivery issues related to workload, poor continuity and parallel coordination of care. Clinical issues related to adequate counselling. Patient factors included a lack of trust, concerns about injections, impact on lifestyle and disposal of needles.

**Conclusion:**

Although resource constraints are likely to remain, district and facility managers can improve supplies, educational materials, continuity and coordination. Counselling must be improved and may require innovative alternative approaches to support clinicians who face high number of patients. Alternative approaches using group education, telehealth and digital solutions should be considered.

**Contribution:**

This study identified key factors influencing insulin initiation in patients with T2DM in primary care. These can be addressed by those responsible for clinical governance, service delivery and in further research.

## Introduction

In South Africa, diabetes is the second leading cause of death and the leading cause of death in women.^1^ The age-adjusted comparative prevalence of diabetes in South Africa is 10.8% (4.2 million people in 2021).^2^ Most people have type 2 diabetes (T2DM) and are dependent on the public sector for healthcare. In the Western Cape, 75% of these patients have poor glycaemic control (glycosylated hemoglobin A1c [HbA1c] > 7%) and a third of these have very poor control (HbA1c > 10%).^3^

There are many factors influencing the poor outcomes for people with diabetes. These include clinical inertia, a lack of patient education and counselling and a failure to initiate insulin when it is required.^4,5,6^ The primary care guidelines in the Western Cape provide two oral agents (glimepiride and metformin) and two forms of insulin (basal and biphasic).^7^

Psychological insulin resistance is defined as resistance to insulin therapy both on the part of patients and healthcare workers.^8^ Patients may be concerned about gaining weight, dose calculations, storage and transportation of insulin, restricted social activities and self-injection.^9^ Clinicians may also be concerned with insulin storage, transportation and dose calculation.^10^ Insulin initiation is often perceived as a last resort, rather than an inevitability, and some clinicians regard it as a failure of lifestyle modification. In addition, a lack of knowledge regarding insulin initiation and intensification is a significant factor.^11,12^

Practitioners’ barriers can include the lack of knowledge and experience with regard to the use of guidelines related to insulin therapy, language barriers and fear of hypoglycaemia.^11^ Patients’ barriers can include beliefs about insulin, non-adherence, a lack of understanding of diabetes, use of traditional herbs, fear of injections and poor socio-economic conditions. System barriers can include inadequate time, a lack of continuity of care and financial constraints.^11,12^ There has been little recent research on this topic in South Africa. The study aimed to explore factors that influence the initiation of insulin in patients with T2DM in primary care facilities in Cape Town, South Africa.

## Methods

### Study design

An exploratory, descriptive qualitative study design was used to explore the factors influencing insulin initiation through semi-structured interviews.

### Study setting

The Cape Town Metro health district is divided into eight sub-districts. The Eastern sub-district has a population of 508 689 people, of which 47% are mixed race and 35% black African. The population dependent on the public sector has no health insurance, has 22% unemployment and mostly live in basic housing or informal settlements.^13^

There were two district hospitals in the area, seven community day centres (CDCs), five clinics and one satellite clinic. There was no 24-h primary care facility. Four of the CDCs had permanent medical officers (MO) (eight in total) and on average each had five clinical nurse practitioners (CNPs), although only one CNP primarily dealt with non-communicable diseases. All CDCs had weekly support from registrars in family medicine and in addition up to six locum MOs were employed. Primary care was mainly offered by nurse practitioners, supported by MOs. According to the primary care guidelines, insulin should be initiated by MOs.^7^ Uncontrolled patients with diabetes are seen monthly for monitoring and adjustment of treatment.

Health services for T2DM included MOs, who were involved in the care of uncontrolled patients and those with complications; these patients were usually seen monthly for monitoring and adjustment of medication. Clinical nurse practitioners followed up uncomplicated patients and renewed prescriptions. Well-controlled patients were placed in chronic clubs, which were also run by CNPs. These patients came to the primary care facilities for review every 6 months and had their HbA1C checked annually. In the case of uncontrolled patients, the HbA1c was meant to be checked every 3 months to monitor improvement and decide on the need for treatment intensification. There were a variety of allied health professionals available in the sub-district, including a dietitian and physiotherapist. They were mainly involved in promoting lifestyle changes for patients with T2DM by advocating for more active lifestyles and improvement in dietary habits.

### Selection of participants

There were four groups of participants selected from primary care facilities. Firstly, permanent MOs with experience of how the facilities operated and of trying to initiate insulin. Secondly, nurse practitioners who had to support patients after insulin was prescribed. Thirdly, patients with T2DM who had the experience of starting insulin, and lastly, the perspective of patients with T2DM who were eligible to start insulin, but had not yet done so. Locum MOs, who only came for 1 to 2 days at a time, as well as patients with type 1 diabetes were excluded.

The intended sample size was 16 participants, with one representative of each group from four different primary care facilities. The final sample size was dependent on saturation of data. Only facilities with permanent MOs were selected, as they were needed to initiate insulin. Facilities were selected to ensure maximum variation in the population served. At each of the facilities, the nurse practitioner in charge of non-communicable disease management and the most senior MOs were selected. Patients, who met the inclusion criteria, were randomly selected from the register of diabetic patients.

### Data collection

Semi-structured interview guides were developed based on previous research.^14,15,16^ The main areas explored with patients on insulin were as follows: experiences with initiating insulin, who was involved in the process, what made the process easier and what they thought could have been carried out differently. For patients not yet on insulin, the questions explored their perceptions of the process and their concerns. For clinicians, the questions explored their experiences with initiating insulin, what had worked well for them in the past and challenges they faced with insulin initiation. The interviews were conducted in English by T.M., were recorded, and lasted from 30 min to 70 min. Fifteen of the interviews were carried out at the primary care facilities and two at the patients’ homes.

### Data analysis

The recorded data were transcribed verbatim and checked for errors. Thematic data analysis used Atlas.ti software and followed the framework method^17^:

- Familiarisation: Immersion in the raw data and transcripts
- Coding index: Codes were derived deductively from the data and organised into categories
- Coding: All transcripts were coded
- Charting: Brought data with the same code and category into one place
- Interpretation: Each chart was read and the data were interpreted to define themes and look for any relationships between themes.

The researcher (T.M.) was a registrar in family medicine working in the sub-district. She provided outreach from the district hospital to one of the selected facilities but did not treat any of the selected patients. She was not an experienced qualitative researcher and was supervised by R.M. Although she had a positive attitude towards initiating insulin, she was careful to remain neutral and open to all perspectives and beliefs in the interview process and analysis. Data from the different groups of participants was triangulated to give a more in-depth understanding.

### Ethical considerations

Ethical clearance was obtained from the Health Research Ethics Council (HREC) – Stellenbosch University (Ethics Reference No.: S20/06/136). Permission was obtained from the Western Cape Department of Health.

## Results

The characteristics of the 17 participants are shown in Table 1 and Table 2. One extra patient was interviewed to ensure data saturation. The following main themes were identified:

- The need for social support at home and work
- Issues with self-administration of insulin
- Inadequate counselling of patients because of human resource constraints
- A lack of educational materials
- Difficulty in retaining patients in care after initiating insulin
- Fear of hypoglycaemia.

**TABLE 1:** Characteristics of patients.

Age (years)	Gender	Taking insulin	Time using insulin	Facility
39	Female	No	-	CDC 1
39	Male	No	-	CDC 2
44	Male	Yes	1 Year	CDC 2
36	Female	Yes	3 Years	CDC 3
54	Female	Yes	1 Month	CDC 4
42	Female	Yes	1 Year	CDC 1
62	Male	Yes	1 Year	CDC 4
26	Female	No	-	CDC 4
60	Female	No	-	CDC 3

CDC, Community Day Centre.

**TABLE 2:** Characteristics of healthcare workers.

Age (years)	Gender	Years of experience	Designation	Facility
49	Female	8	CNP	CDC 3
37	Female	6	CNP	CDC 2
48	Female	5	CNP	CDC 1
43	Female	9	CNP	CDC 4
33	Female	1	MO	CDC 4
29	Female	2	MO	CDC 3
53	Female	5	MO	CDC 2
45	Male	5	MO	CDC 1

MO, medical officer; CNP, clinical nurse practitioner; CDC, community day centre.

### The need for social support at home and work

All participants acknowledged the importance of good social support, particularly for older adults. Clinicians saw the value of educating family members as well as patients:

‘A lot of the times or most of the times it’s an elderly person, and I am kind of concerned if the person is understanding what I’m saying, if they are able to relay the message to somebody that can assist them. So, it would be easier if there is somebody coming with the patient.’ (Medical Officer, 4 years’ experience, CDC 1)

The patients emphasised the support and encouragement that they received from various family members who might also be taking insulin or could help them adhere to the treatment. Food security was a concern for some of the patients, especially those who were unemployed, and this made them reluctant to start insulin. Employed patients stated that their employers were aware of their need to take insulin, but several preferred to inject themselves before or after work. This preference was related to issues with regard to storage of insulin, having a safe and private place to inject and being able to discard needles. Shift work could complicate taking insulin, while having access to an occupational health nurse could help:

‘Because she has in her mind, “Doctor, I can’t use insulin. I’m not going to use those injections. I think it’s the food I’m eating” … So, they bring more social issues to support the poor [glycaemic] control.’ (Medical Officer, 4 years’ experience CDC 1)

### Issues with self-administration of insulin

One of the factors mentioned by all was the reluctance of patients to inject themselves and a lack of confidence. Clinicians stated that although they demonstrated how to inject insulin, patients remained concerned and did not understand the process fully:

‘Okay try, they give also the needles and then I inject myself at night. But one of the needles, when I use it, it just breaks … I was so shocked about that.’ (42-year old patient on insulin, CDC 1)

Accurate measurement of doses was a concern. Patients who used pen sets stated that they were easier to use, but these were only issued to older patients or visually impaired. Current guidelines recommend issuing patients with glucometers to monitor their glucose and adjust their dose. Most patients had no difficulty with using the meters; the main challenge was the supply of enough test strips:

‘Another small little challenge is the strips, there is not always enough. Especially for the twice, the Actraphane. We do have a few patients on Actrapid as well and so that is just like a challenge that I also like to complain about.’ (Medical Officer, 2 years’ experience, CDC 3)

‘They gave me … the needles and the insulin and the machines. And the papers, for the book so that I must read it and then I must know it, when it is high, it is this number and when it is normal it is this number.’ (42-year old patient on insulin, CDC 1)

Patients not yet initiated on insulin had many concerns, particularly regarding injection times and meals:

‘So, I asked, when I work nightshift can I take it like [in the] morning because I’m working. You understand?’ (44-year old patient on insulin, CDC 2)

Clinicians found that patients preferred the basal insulin over the biphasic insulin. Patients were aware that insulin should be stored in a fridge but were uncertain about how long it could be kept out, particularly when they were travelling long distances.

### Inadequate counselling of patients because of human resource constraints

Respondents felt that there were inadequate human resources to cope with the needs of people with diabetes. Practitioners had to limit the duration of consultations in order to get through the workload and did not have capacity to properly counsel or educate patients:

‘Because with our numbers we see 40 patients a day it will be difficult to give 20 minutes or 15 minutes per patient, it would not work.’ (Medical Officer, 4 years’ experience, CDC 1)

At two of the clinics, they previously had dedicated diabetic clinic days, and this had helped with initiating insulin, as the doctor had more time. However, these initiatives had ended when the doctors left:

‘We allocated Tuesdays as diabetic day and that’s why when you come on Tuesdays you see a lot of diabetics and we selected randomly who had uncontrolled HbA1c celevated, to be seen by the registrar and take time because they were booked, per day 10 or 15 [*patients*].’ (Medical Officer, 4 years’ experience, CDC 1)

Patients were hesitant to ask questions when they were aware of the queue and the many patients waiting. Patients might get only one or two appointments with a dietitian or physiotherapist after diagnosis. Patients frequently returned to the nurse to explain further after seeing the doctor. The coronavirus disease 2019 (COVID-19) pandemic further reduced the opportunities for education and counselling. Although all the MOs were aware of the primary care guidelines, there was a lack of comprehensive and ongoing education and counselling. This meant that patients were poorly informed from the time of diagnosis and did not anticipate the need for insulin. Two of the patients stated they had only realised they were started on insulin when they found it in their packets of medication at the pharmacy:

‘But the only thing I was not happy was, I was told you’re going to be put now on insulin and stuff like that, and then we get given our paperwork … After we received the medication, we then go home.’ (36-year old patient on insulin, CDC 4)

In some cases, after insulin was dispensed, the packets were found in bins outside the clinic. This implied a lack of counselling and shared decision making on starting insulin. Patients stated it would be better for someone to show them how to use insulin rather than just telling them:

‘Physically show them because sometimes you need to show somebody how to do it and not just assume that they would be able to do the right thing. Because it can do more harm to the person to not know.’ (36-year old patient on insulin, CDC 4)

### A lack of educational resources

Clinicians commented on the lack of posters and handouts to educate patients on using insulin. Some clinicians were aware of group education programmes and thought these could include sessions on starting insulin. Even when there were pamphlets available, some patients struggled because of visual impairment:

‘At the moment we don’t have a lot of literature for the patient.’ (Medical Officer, 2 years’ experience, CDC 2)

‘But the sister used to have posters where she will show them where to inject when she’s giving the talks.’ (Clinical Nurse Practitioner, 6 years’ experience, CDC 2)

### Difficulty retaining patients in care after initiating insulin

After starting insulin some patients did not return, maybe because they did not understand or relocated to a different area. There was no system to trace patients, who did not show up for their appointments, and addresses in the patients’ records were often incorrect:

‘So, it really is up to them. We do basically throw them under the bus by saying look, this is your machine, this is your this, this is this, do that, do that, come back if you need it. They don’t come back. Oh, they really don’t come back.’ (Medical Officer, 2 years’ experience, CDC 3)

After initiating insulin, MOs stated they usually made an appointment for 2 weeks, but if the patient did not come, then follow up would be the usual 4 weeks. Everyone expressed dissatisfaction with the lack of continuity of care. For nurses, it was the challenge of working with different MOs who followed different guidelines. Patients preferred to be seen by the same clinician.

### Fear of hypoglycaemia

Respondents worried about hypoglycaemia, particularly in the elderly and those with multi-morbidity. Patients struggled with food security and the risk of injecting insulin without an adequate meal:

‘It’s hypoglycaemia, because that’s what scares me personally, if the patient doesn’t understand.’ (Medical Officer, 4 years’ experience, CDC 1)

All the clinicians interviewed stated that they explained about the risk of hypoglycaemia. Interestingly, they could only remember two patients with hypoglycaemia, both of whom had chronic renal impairment.

## Discussion

**FIGURE 1:**
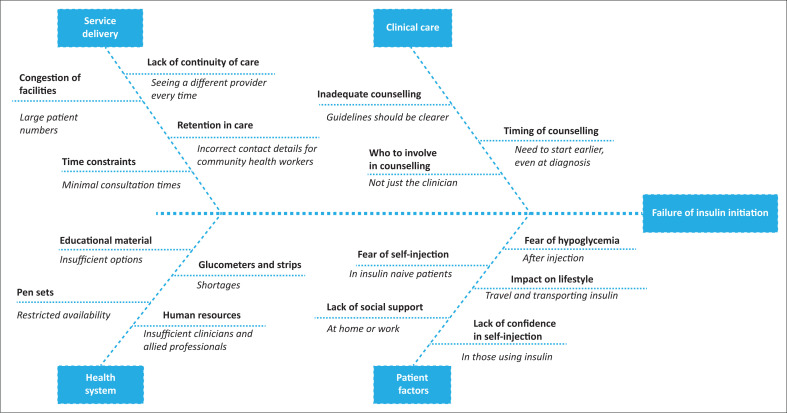
Summary of the key findings.

Figure 1 summarises the key findings in terms of the health system, service delivery, process of clinical care and patient-related factors.

In terms of service delivery, congestion, queues, high workload and brief consultations are a challenge at most facilities in the public sector.^18^ More resources have been put into human immunodeficiency virus (HIV) and tuberculosis (TB) care than diabetes, and for these patients there are established mechanisms for retaining patients in care via community health workers (CHWs).^19,20^ This type of commitment to retain patients with T2DM in care and support initiation of insulin in the community is potentially part of the CHWs’ scope of practice, but is not yet fully implemented.^21^ A lack of continuity of care has also been recognised as a weakness of service delivery for local primary care.^11,18^ Continuity of care is a core component of primary care and has been linked to better patient outcomes.^18^

In terms of clinical care, all clinicians interviewed were familiar with current guidelines on management of T2DM.^7,22^ This was in contrast to other studies, where the lack of familiarity was one of the barriers to insulin initiation.^15,16^ The guidelines are clear on when insulin should be initiated, but do not provide guidance on the content or timing of counselling. Previous research has placed emphasis on counselling patients about the progressive nature of T2DM and the likelihood of progressing to insulin therapy.^7,15,16^

In terms of the health system, centralised chronic medicine dispensing for ‘stable’ patients via alternative pick-up-points has improved the supply of medication and test strips.^23^ There is a need to look at mechanisms for the supply of glucometers, education in their use, and enough test strips. More detailed patient education materials need to be available to support initiation of insulin. While educational materials and improved knowledge alone are unlikely to be sufficient to enable initiation of insulin, having such materials available as adjuncts can reinforce the key messages given during counselling.^24^

In terms of patient factors, the patients mostly understood the value of insulin, but anxiety, a lack of confidence, and worries about the impact on lifestyle, were inadequately addressed and as observed elsewhere there was a lack of support for starting insulin.^14,16,25^ Having a family member or friend who already used insulin successfully can facilitate successful initiation.^15,16,25^ Fear of hypoglycaemia was noticed as a barrier in multiple studies on insulin initiation, and all clinicians in this study were concerned about this.^12,14,26^ Clinicians may worry more about this than patients.^12,16^

Although there was a possibility of social desirability bias, this was mitigated by the fact that the researcher was unknown to the patients and most of the staff. Respondents appeared to speak freely about their negative as well as positive experiences. The study was conducted during the COVID-19 pandemic and perspectives may have been influenced by changes in service delivery. Data saturation was considered to have been reached as there were no new themes emerging from the last two interviews. The findings discussed in this article should be transferable to primary care facilities across the Metro Health Services (MHS), which serve similar populations and provide similar services. The primary care context and patients are like other public sector primary care contexts in South Africa.

Alternative approaches to patient education and counselling should be explored, to overcome the constraints of a high workload and brief consultations. Group empowerment and training (GREAT) for diabetes has already been implemented in this setting and additional bespoke sessions on insulin initiation could be added.^27^ Another innovation during COVID-19 was telehealth support for people with poorly controlled T2DM and affected with COVID-19.^28^ A similar service could support patients during the initiation of insulin.

Digital solutions to provide information and support may also be useful. In Cape Town, a WhatsApp Chatbot has been shown useful in diabetes education.^29^ In Tshwane, a nurse-driven application has supported people as they start insulin with some success.^30^

Guidelines should specify the timing and broad content of counselling for patients regarding the use of insulin. The likelihood of requiring insulin at a later stage should be discussed soon after diagnosis.^15,16,25^ Facility organisation and management should enable continuity of care.

Alerts on patients that are not retained in care or do not collect their insulin should be developed. These could come from the pharmacy and CHWs would be able to follow up at home. Community health workers and nurse coordinators could provide additional support and adherence counselling. Care must be taken to ensure the health information system contains the correct addresses and contact details.

Educational materials should be improved and tailored to the type of education and counselling used, such as group education, telehealth or support via apps. Supply chain management should also be improved so that test strips do not run out and glucometers are available.

Further research is needed to design, develop and evaluate alternative approaches to patient education, counselling and support as suggested here. Research may also evaluate the implementation of more adherence support via CHWs.

## Conclusion

The main barriers to initiation of insulin were time constraints and high workload in clinical practice, poor continuity and retention in care, inadequate education, counselling and support, fears of self-injection and concerns of the impact on lifestyle. Alternative approaches to patient education and counselling, using group approaches, telehealth or digital technology, should be designed, developed and evaluated further.
